# Supplementary material for: Deletion of podocyte Rho-associated, coiled-coil-containing protein kinase 2 protects mice from focal segmental glomerulosclerosis
Source: Commun Biol. 2024 Apr 2;7:402. doi: 10.1038/s42003-024-06127-3 (PMC10987559; doi:10.1038/s42003-024-06127-3)
Supplement: Supplementary file 2 — Supplementary Information [file 42003_2024_6127_MOESM2_ESM.pdf]

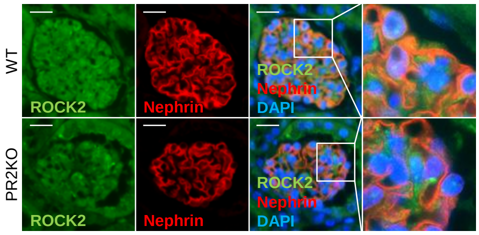

Supplementary Fig. 1: Generation of podocyte-specific ROCK2-knockout mice. Immunolabeling of ROCK2 and the expression of nephrin in kidney sections obtained from WT and PR2KO mice. The scale bar at the left top represents 10 μm.

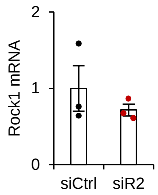

Supplementary Fig. 2: Characterization of ROCK2-deficient podocytes. The mRNA levels of ROCK1 in podocytes treated with siRNA against ROCK2 (n = 3). Data represent the mean ± s.e.m.

Fig. 1c

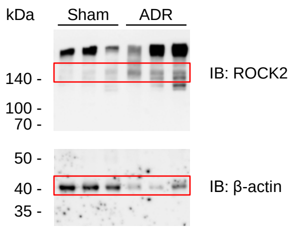

Fig. 1g

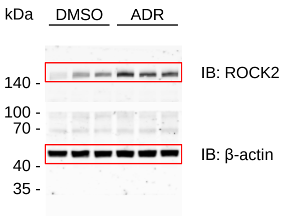

Supplementary Fig. 3: Unedited images. Unprocessed blot for Fig. 1c and g are presented.

Gene	Organism	GenBank Accession	Forward (5' to 3')	Reverse (5' to 3')
Actb	Mus musculus	NM_007393	GTGACGTTGACATCCGTAAAGA	GCCGGACTCATCGTACTCC
Rock2	Mus musculus	NM_009072	GGTTTACAGATGAAAGCGGAAGA	GTGATGCCTTATGACGAACCAA
Tgfb1	Mus musculus	NM_011577	CCACCTGCAAGACCATCGAC	CTGGCGAGCCTTAGTTTGGAC
Pai1	Mus musculus	NM_008871	TCTGGGAAAGGGTTCACTTTACC	GACACGCCATAGGGAGAGAAG
Acta2	Mus musculus	NM_007392	CCCAGACATCAGGGAGTAATGG	TCTATCGGATACTTCAGCGTCA
Fn1	Mus musculus	NM_010233	ATGTGGACCCCTCCTGATAGT	GCCCAGTGATTTCAGCAAAGG
Rgs2	Mus musculus	NM_009061	GAGAAAATGAAGCGGACACTCT	GCAGCCAGCCCATATTTACTG

Supplementary Table 1: Primer sequences.
