# Supplementary material for: Deletion of podocyte Rho-associated, coiled-coil-containing protein kinase 2 protects mice from focal segmental glomerulosclerosis
Source: Commun Biol. 2024 Apr 2;7:402. doi: 10.1038/s42003-024-06127-3 (PMC10987559; doi:10.1038/s42003-024-06127-3)
Supplement: Supplementary file 3 — Description of Additional Supplementary Files [file 42003_2024_6127_MOESM3_ESM.pdf]

Description of Additional Supplementary Files

File name: Supplementary Data 1
Description: Numerical source data for graphs in the manuscript.
