# Supplementary material for: Deletion of podocyte Rho-associated, coiled-coil-containing protein kinase 2 protects mice from focal segmental glomerulosclerosis
Source: Commun Biol. 2024 Apr 2;7:402. doi: 10.1038/s42003-024-06127-3 (PMC10987559; doi:10.1038/s42003-024-06127-3)
Supplement: Supplementary file 5 — Reporting Summary [file 42003_2024_6127_MOESM5_ESM.pdf]

nature portfolio

Corresponding author(s): Keiichiro Matoba

Last updated by author(s): Mar 13, 2024

# Reporting Summary

Nature Portfolio wishes to improve the reproducibility of the work that we publish. This form provides structure for consistency and transparency in reporting. For further information on Nature Portfolio policies, see our Editorial Policies and the Editorial Policy Checklist.

## Statistics

For all statistical analyses, confirm that the following items are present in the figure legend, table legend, main text, or Methods section.

| n/a | Confirmed | |
|---|---|---|
| ☐ | ☒ | The exact sample size (*n*) for each experimental group/condition, given as a discrete number and unit of measurement |
| ☐ | ☒ | A statement on whether measurements were taken from distinct samples or whether the same sample was measured repeatedly |
| ☐ | ☒ | The statistical test(s) used AND whether they are one- or two-sided<br>*Only common tests should be described solely by name; describe more complex techniques in the Methods section.* |
| ☐ | ☒ | A description of all covariates tested |
| ☐ | ☒ | A description of any assumptions or corrections, such as tests of normality and adjustment for multiple comparisons |
| ☐ | ☒ | A full description of the statistical parameters including central tendency (e.g. means) or other basic estimates (e.g. regression coefficient) AND variation (e.g. standard deviation) or associated estimates of uncertainty (e.g. confidence intervals) |
| ☐ | ☒ | For null hypothesis testing, the test statistic (e.g. *F*, *t*, *r*) with confidence intervals, effect sizes, degrees of freedom and *P* value noted<br>*Give P values as exact values whenever suitable.* |
| ☒ | ☐ | For Bayesian analysis, information on the choice of priors and Markov chain Monte Carlo settings |
| ☒ | ☐ | For hierarchical and complex designs, identification of the appropriate level for tests and full reporting of outcomes |
| ☐ | ☒ | Estimates of effect sizes (e.g. Cohen's *d*, Pearson's *r*), indicating how they were calculated |

*Our web collection on statistics for biologists contains articles on many of the points above.*

## Software and code

Policy information about availability of computer code

| | |
|---|---|
| Data collection | N/A |
| Data analysis | Histological findings were quantified by the ImageJ software program (National Institutes of Health, Bethesda, MD). |

For manuscripts utilizing custom algorithms or software that are central to the research but not yet described in published literature, software must be made available to editors and reviewers. We strongly encourage code deposition in a community repository (e.g. GitHub). See the Nature Portfolio guidelines for submitting code & software for further information.

## Data

Policy information about availability of data

All manuscripts must include a data availability statement. This statement should provide the following information, where applicable:

- Accession codes, unique identifiers, or web links for publicly available datasets
- A description of any restrictions on data availability
- For clinical datasets or third party data, please ensure that the statement adheres to our policy

RNA-Seq data are available at https://doi.org/10.6084/m9.figshare.23276111.v1

## Research involving human participants, their data, or biological material

Policy information about studies with human participants or human data. See also policy information about sex, gender (identity/presentation), and sexual orientation and race, ethnicity and racism.

| | |
|---|---|
| Reporting on sex and gender | N/A |
| Reporting on race, ethnicity, or other socially relevant groupings | N/A |
| Population characteristics | N/A |
| Recruitment | N/A |
| Ethics oversight | N/A |

Note that full information on the approval of the study protocol must also be provided in the manuscript.

# Field-specific reporting

Please select the one below that is the best fit for your research. If you are not sure, read the appropriate sections before making your selection.

☒ Life sciences ☐ Behavioural & social sciences ☐ Ecological, evolutionary & environmental sciences

For a reference copy of the document with all sections, see nature.com/documents/nr-reporting-summary-flat.pdf

# Life sciences study design

All studies must disclose on these points even when the disclosure is negative.

| | |
|---|---|
| Sample size | The sample size was determined based on literature. |
| Data exclusions | No data were excluded from the analysis. |
| Replication | The measures were described in figure legends. |
| Randomization | Mice were randomly divided into experimental groups. |
| Blinding | For each animal, different investigators were involved. An investigator administered ADR or SLx-2119. This investigator was the only person aware of the treatment group allocation. Other investigators were unaware of the treatment. |

# Reporting for specific materials, systems and methods

We require information from authors about some types of materials, experimental systems and methods used in many studies. Here, indicate whether each material, system or method listed is relevant to your study. If you are not sure if a list item applies to your research, read the appropriate section before selecting a response.

### Materials & experimental systems

| n/a | Involved in the study | |
|---|---|---|
| ☐ | ☒ | Antibodies |
| ☐ | ☒ | Eukaryotic cell lines |
| ☒ | ☐ | Palaeontology and archaeology |
| ☐ | ☒ | Animals and other organisms |
| ☒ | ☐ | Clinical data |
| ☒ | ☐ | Dual use research of concern |
| ☒ | ☐ | Plants |

### Methods

| n/a | Involved in the study | |
|---|---|---|
| ☒ | ☐ | ChIP-seq |
| ☒ | ☐ | Flow cytometry |
| ☒ | ☐ | MRI-based neuroimaging |

## Antibodies

| | |
|---|---|
| Antibodies used | Anti-ROCK2 antibody (#ab71598; Abcam, Cambridge, UK)<br>Anti-nephrin antibody (#BP5030; OriGene, Rockville, MD)<br>Wilms' tumor 1 (WT1) antibody (#ab89901; Abcam) |

| | |
|---|---|
| Validation | Antibody validation information is available on the manufacturer's website. |

## Eukaryotic cell lines

Policy information about cell lines and Sex and Gender in Research

| | |
|---|---|
| Cell line source(s) | Conditionally immortalized murine podocytes (E11) |
| Authentication | A conditionally immortalized murine podocyte cell line (E11) was obtained from Cell Line Services, but was not further authenticated after purchase. |
| Mycoplasma contamination | Mycoplasma-free E11 podocytes were propagated in 10 U/mL murine interferon-γ at 33°C and then differentiated by culture for 10-14 days at 37°C in the absence of interferon-γ. |
| Commonly misidentified lines (See ICLAC register) | No cell lines used in this study were found in the database of commonly misidentified cell lines that is maintained by ICLAC and NCBI BioSample. |

## Animals and other research organisms

Policy information about studies involving animals; ARRIVE guidelines recommended for reporting animal research, and Sex and Gender in Research

| | |
|---|---|
| Laboratory animals | In the first set of experiments, 8-week-old C57BL male mice are used. In the second set of experiments, 6-week-old male C57BL/6J mice were used as described in methods section. |
| Wild animals | N/A |
| Reporting on sex | Only male mice were used for our studies to reduce variability in weight gain and metabolism due to gender. |
| Field-collected samples | N/A |
| Ethics oversight | Studies dealing with animal use were conducted under protocols approved by the Committee on Ethical Animal Care and Use of the Jikei University School of Medicine. |

Note that full information on the approval of the study protocol must also be provided in the manuscript.
